# Psychodynamic interventions in palliative care: Cui bono?

**DOI:** 10.1017/S1478951525101041

**Published:** 2025-11-03

**Authors:** Friedrich Stiefel, Céline Bourquin, Laurent Michaud

**Affiliations:** Psychiatric Liaison Service, Lausanne University Hospital and University of Lausanne, Lausanne, Switzerland

About 2 years ago, we published in this journal a critical personal reflection paper entitled “Positive psychology interventions in palliative care: Cui bono?” (Stiefel et al. 2024). We referred to a systematic review (Van Zyl et al. 2024), which summarized different theoretical and conceptual criticisms addressed towards positive psychology. In our critical stance, we expanded the discussion by introducing clinical arguments that had been absent from previous debates, leading us to express important reservations about the systematic implementation of positive psychology interventions in palliative care. Specifically, we raised several apprehensions about the increasing number of interventions targeting positive subjective states, feelings, or experiences such as optimism, hope, post-traumatic growth, or gratitude to improve patients’ quality of life. These apprehensions questioned certain assumptions such as: why should subjective states, not in themselves pathological or dysfunctional, be targeted? Why should existential experiences fall into the realm of psychology? How do we assure that directive positive psychology interventions do not hamper the therapeutic relationship, which is based on the need to “meet patients where they are” and not “where we want them to be”? What space is provided to express negative emotions that coexist with positive ones? Finally, we asked “Cui bono?”: Do these interventions serve the needs of patients or those of clinicians? Based on this critical stance as liaison psychiatrists with a psychodynamic orientation (FS and LM) and as a social scientist embedded in liaison psychiatry (CB), we considered that the same question could also be applied to psychodynamic interventions in palliative care. What purpose do they serve? What do we have to offer in situations of existential distress? Here is our answer.

## Psychodynamic interventions in palliative care: Basic assumptions

Psychodynamic-oriented interventions are distinguished from other psychotherapeutic approaches by a set of fundamental assumptions (Stiefel and Bernard 2008); which we will summarize in the following illustrating their relevance in the palliative care setting by means of short and emblematic examples and a clinical vignette. Based on Freud’s work, object relation and attachment theory, as well as self-psychology, psychodynamic interventions intend to enhance self-understanding, insight into one’s relations with others, and autonomy. The following theoretical assumptions underlie psychodynamic psychotherapy. First, unconscious forces shape thoughts, emotions, and behaviors (i). This can be observed in the palliative care setting when unconscious separation anxiety shared between patients and clinicians lead to collusive interactions, which may not only provoke psychological suffering (Stiefel et al. 2017), but also negatively impact care (Deliyanidis et al. 2024). Second, human development and associated difficulties influence the way we experience others and the world in later stages of life (ii). For example, intense rivalry with a younger brother and competition for parental love, may reappear as an adult patient by a tendency to grab attention and to make special requests towards physicians (Stiefel and Michaud accepted for publicationin press). Third, the psyche is dynamically organized by the interacting forces of the ego, the id, and the superego (iii). Harsh superego demands in patients unable to maintain their duties due to progressive illness may then lead to depressive states colored by feelings of guilt, which remain incomprehensible for the concerned patient (Busch 2009). Fourth, the psychic equilibrium is maintained by defense mechanisms (iv), which modulate emotional resonance when facing turmoil in the inner and outer worlds. The relevance of defenses in end-of-life situations has been commented by Weisman, who observed that patients cope if they can, deny if they must and get psychotic when they are forced to (Weisman 1979). And lastly, unresolved relational issues and conflicting intrapsychic forces (e.g., desire for closeness and fear of dependency), re-enacted as transference in the relationships with others (v), can be identified and worked through, liberating the patient from constraints of the past. Such transferential reactions become especially salient as the disease progresses and end-of-life approaches, as illustrated by the following clinical vignette.

*A 55-year-old businessman, suffering from metastatic lung cancer, was referred to one of the co-authors (FS) for anxiety and agitation. The anxious agitation was due to a threat, which was initially not accessible to the patient’s consciousness ((i) = first theoretical assumption described above), and thus not identifiable. By diving deeper into the history of the patient’s early development (ii), marked by parental neglect, it became clear that the threat was not provoked by the cancer per se, but related to increasing dependency on the medical apparatus. From a dynamic point of view, the patient had harsh superego demands and very performant ego functions (iii), enabling him to realize a successful career as a self-made man. Most important, this career allowed him to achieve an independent position, without a need to rely on others. His privileged defense mechanisms facing death and thus radical separation from his family were intellectualization and rationalization (iv), both mechanisms, already operating* during his professional career. *These defenses manifested themselves for example when he stated that billions of humans died before him and billions will die after him… a statement he often repeated and which troubled his wife, since she felt that her husband somehow denied their longtime relationship. The unresolved issue (v) over dependency wishes and fear of letting go and being cared for by others, which could be understood as his basic conflict, emerged in his struggle to adopt the patient role. Once these elements became more conscious and addressed, the patient – true to himself – decided to “start a new project”: to become less preoccupied with autonomy. He searched and found a way to serenity, motivated by the desire to leave a cherished memory for his family; a memory of a father and husband courageously facing the inevitable.*

## Psychodynamic interventions: An interactional perspective

As illustrated in this clinical vignette and in contrast to positive psychology interventions targeting healthy states like hope or gratitude, psychodynamic interventions have indications, based on the presence of psychological suffering. Another key element is that the patients are the focus of the intervention, but attention is also brought to the clinicians and their countertransference. Recognition of clinicians’ own positive and negative emotions, thoughts and experiences with patients are considered as important means preventing that their own needs predominate over those of the patient. This implies that the clinical encounter is viewed as a co-construction and that the focus of attention includes clinicians and their unresolved issues, desires, preoccupations, which contribute to the interactional dynamics. Clinicians’ own psychotherapy is thus an essential element of becoming a therapist. This is especially important in settings where the clinical encounter is characterized by high relational and emotional intensity such as palliative care. Indeed, the existential threat concerns not only the patient but also resonates in the clinician. Clinicians must therefore develop insight into how they relate to the existential experience of vulnerability, finitude or solitude (Stiefel and Michaud accepted for publication) that emerges in life-threatening diseases. The psychodynamic approach, which considers countertransference, thus places particular emphasis on distinguishing between clinicians’ and patients’ needs, thereby consistently addressing the question “Cui bono.”

## Psychodynamic interventions and existential distress

In psychodynamic interventions (Stiefel and Bernard 2008), the clinician first attempts to situate and encounter the patients “where they are,” which implies to address and at times first intensify their suffering. This can be uncomfortable, for both patients and clinicians, but also brings them together. Sharing difficult emotions, thoughts, stories and experiences is a way to relate to others and thus to reduce feelings of loneliness or alienation. Psychodynamic interventions do not target existential experiences per se; rather, they focus on how psychological suffering is induced by or shapes the experience of existential threat. In other words, the existential fact itself is not the target of psychodynamic interventions, but they may elucidate how psychological functioning articulates with the experience of the existential dimensions of illness. We have previously illustrated (Stiefel and Michaud accepted for publicationin press) the emergence of existential dimensions in the psychotherapy of a patient with advanced cancer, corresponding to the observations of Karl Jaspers (Jaspers 1986): recognition of human vulnerability and finitude; the quest for sense-making when life is threatened; and the experience that we – as individuals – are somehow lonely and separated from others, that nobody can carry our destiny, take off the burden of being ill, or die at our place. In case of existential distress, the psychodynamic perspective seeks to understand how patients uniquely relate to these dimensions and how distress is provoked.

The clinical vignette presented above illustrated how and why vulnerability was so threatening for this patient and how the approaching finitude and imminent separation from his family triggered defense mechanisms that mitigated his anxiety of separation. Under therapy and once the agitation lowered, the patient found a way to make sense of his (remaining) life. The loneliness of enduring a terminal illness, probably recalling feelings experienced during his development, was never addressable in therapy. However, he reported that from times to times he felt as if someone was pulling the rug out from under his feet. This feeling of loneliness, which erupted from time to time, was too painful to be elaborated and worked through; it can be considered as fear of breakdown (Winnicott 1974).

We observe that a certain dialectic exists regarding these existential dimensions of disease. Of course, we are vulnerable, especially when falling ill; yet we can rely on medicine and other sources of support to help mitigate this vulnerability. Indeed, death is the unthinkable, but from time to time we may accept to face it, at least for a short time, and thus reduce its terror. To suffer and die might feel absurd; nonetheless, some can find a sense in what we endure. This sense may relate to supra-individual, spiritual, philosophical or religious experiences, or take a more biographical-psychological form as illustrated by the patient in the vignette. And finally, the loneliness of the human existence is undeniable; however, as Heidegger stated “Sein ist immer auch Mitsein” (being is always also being with) (Rentsch 2015), and sharing our experiences with others can help alleviate some of this loneliness. Especially when this dialectic of the existential dimensions has fallen out of balance, a psychodynamic understanding of how this imbalance is fueled by the psychological experience of the patient may foster meaning and restore a sense of appeasing control (Viederman 1983).

## A psychodynamic understanding of loss

A psychodynamic understanding in the palliative care setting can also contribute to provide a specific perspective on loss. Indeed, the clinic of palliative care is profoundly shaped by the experience of loss. Many patients have already experienced multiple losses (e.g., of health, carefreeness, bodily parts, strengths, hope for a cure) and ultimately face the loss of life and of the loved ones. On the other hand, clinicians working in palliative care experience the loss of their patients, with whom they establish intense relationships.

Loss, and how it is experienced and practiced is the focus of a book by Reckwitz’ (Reckwitz 2024):“Loss – An Essential Problem of Modernity.” Reckwitz provides a sociological perspective on the transformation of the experiences of loss over the centuries. According to Reckwitz, religion and later the belief in never-ending progress, somehow mitigating the experience of loss are less operant nowadays, leaving the individual somewhat “helpless” in the face of loss. However, as Reckwitz further elaborates, new attempts to attenuate losses have emerged. We consider as examples of these attempts in the medical setting, the strive for post-traumatic growth in cancer survivors, often celebrated as “he-” or “sheroes” (Stiefel and Bourquin 2018). Moreover, practices discussed in our previous paper on positive psychology interventions (Stiefel et al. 2024), aiming to promote hope or gratitude in the face of loss of life, somehow echoing religious practices, can also be considered as practices attenuating experience of loss. Interestingly, Reckwitz suggests that psychoanalytic theory is a key to more adequately face loss. He observes that from a psychoanalytic perspective our development is shaped by loss (the prize of discovery and autonomy is the “loss” of the parents). Moreover, each new stage of man (Erikson 1963) involves loss, leaving the prior stage behind (e.g., childhood when entering adulthood, of professional identity when retiring). Reckwitz underlines that a psychoanalytic understanding does not avoid the negative and tragic and acknowledges loss. Acknowledging loss creates space for vulnerability and a salvatory attitude of protection of ourselves and our fellow humans.

## The shortcomings and pitfalls of psychodynamic interventions

As for any therapeutic intervention, indications for psychodynamic therapy need to be considered. These include the severity of psychopathology – some situations call for alternative approaches, such as relaxation or medication – but also the patients’ preferences, as well as their motivation to explore underlying meanings of distress and to work through emotionally challenging issues. Psychodynamic interventions can be tailored along a spectrum from more supportive to more exploratory or interpretative aims (Stiefel and Bernard 2008), depending on the patient’s needs. Unexperienced clinicians may at times favor an exploratory approach when support is needed, with the consequence that patients become too distressed or terminate therapy. Indeed, in our study evaluating a systematic offer of psychodynamic therapy in the oncology setting, about half of the patients preferred sharing experiences over focusing on specific unresolved issues (Krenz et al., 2014; Ludwig et al., 2014). One fourth expressed a desire to work through personal issues, and another fourth to explore relational difficulties with significant others. This highlights the importance of adjusting the approach to the patients’ needs rather than pursuing one’s own interest.

Moreover, in applying psychodynamic concepts, there can sometimes be a tendency to understand developmental experiences as causal factors of current distress. Yet psychodynamic theory is epistemologically more closely aligned with “understanding” – the search of meaning – than with the “explaining” of the natural sciences seeking to establish causal relationships. A reductionistic view on the consequences of development falls short and can provoke misunderstandings between patients and their significant others.

Another pitfall of psychodynamic approaches is the neglect of systemic determinants of psychological suffering by clinicians focusing on the intrapsychic. Stressors exceeding adaptive capacities exist and social determinants provoking psychopathology cannot be ignored. Neglecting the systemic perspective can lead to feelings of alienation and loneliness.

Finally, psychodynamic theory, like any theoretical framework, can be used defensively to avoid a real encounter with patients; this is especially a risk when treating patients in existential distress, whose struggles resonate in those who care for them. Psychodynamic explanations then serve to counteract feelings of powerlessness of the clinician. This is the reason why regular supervisions in which the question “Cui bono” is raised is both necessary and beneficial for clinicians, and indirectly, patients.

## Conclusions

We consider that psychodynamic interventions in palliative care can contribute to decrease psychological and existential suffering of patients articulating the psychological and existential dimensions of disease. A psychodynamic understanding requires clinicians’ insight in own needs, fears and desires, and provides a key to reflect on the relationship that we, our patients, medicine and society establishes with loss. To come back to our initial question “Psychodynamic interventions in palliative care: Cui bono?,” we consider that a psychodynamic understanding of the clinics of palliative care and the psychodynamic interventions for patients struggling with existential dimensions are relevant and effective to alleviate distress.
